# Evaluating a Glioma Transcriptomic Signature Against a Clinical Reference Model and a Random-Signature Null Distribution: A Leakage-Controlled Internal Audit and a Survey of the Field

**DOI:** 10.3390/diagnostics16172803

**Published:** 2026-08-31

**Authors:** Seyma Yasar, Burak Yagin, Sarah A. Alzakari, Amal K. Alkhalifa, Fahaid Al-Hashem, Abedelmalek Kalefh Tabnjh

**Affiliations:** 1 Department of Biostatistics and Medical Informatics, Faculty of Medicine, Inonu University, 44280 Malatya, Türkiye; seyma.yasar@inonu.edu.tr; 2 Department of Computer Sciences, College of Computer and Information Sciences, Princess Nourah bint Abdulrahman University, P.O. Box 84428, Riyadh 11671, Saudi Arabia; saalzakari@pnu.edu.sa (S.A.A.); akalkalifh@pnu.edu.sa (A.K.A.); 3 Department of Physiology, College of Medicine, King Khalid University, Abha 61421, Saudi Arabia; fahaid999@yahoo.com; 4 Department of Cariology, Institute of Odontology, The Sahlgrenska Academy, University of Gothenburg, 40530 Gothenburg, Sweden; 5 Department of Applied Dental Sciences, Faculty of Applied Medical Sciences, Jordan University of Science and Technology, Irbid 22110, Jordan; 6 Department of Pathology, Saveetha Medical College and Hospital, Saveetha Institute of Medical and Technical Sciences, Chennai 602105, India

**Keywords:** glioma, survival analysis, gene expression, clinical prediction model, calibration, external validation, data leakage

## Abstract

**Objective:** The glioma prognostic literature contains a large number of transcriptomic risk signatures, yet whether these signatures add measurable information beyond a clinical model containing grade and molecular markers is rarely tested. We pursued three objectives jointly: to evaluate a leakage-controlled signature in terms of both discrimination and calibration; to test it against a clinical reference model and against a null distribution of random signatures; and to quantify the reporting practice of the field. **Methods:** The CGGA mRNAseq_693 cohort (*n* = 404, 209 deaths) served as development, the CGGA mRNAseq_325 cohort (*n* = 222, 138 deaths) as independent internal validation, and the TCGA lower-grade glioma and glioblastoma cohorts (*n* = 664, 247 deaths) as external validation. A univariate Cox score test was applied to 17,544 genes with false discovery rate control by the Benjamini–Hochberg procedure, and an elastic-net penalised Cox model was fitted on the top 200 candidate genes. Every model-building operation-imputation, scaling, candidate pool, and penalty selection was confined to the development cohort, and the final model was locked. Three arms were compared: clinical only, transcriptomic only, and combined. Calibration was quantified by the integrated calibration index derived from a smoothed calibration curve. In addition, the abstract-level reporting content of 515 glioma signature records indexed in Web of Science was analysed. **Results:** The signature made no measurable contribution beyond the clinical reference model. In the independent internal validation cohort the clinical model reached a concordance index of 0.800 (95% CI 0.767–0.831) and the signature 0.801 (0.769–0.832); the paired bootstrap difference was indistinguishable from zero (Δ = +0.001; 95% CI −0.032 to +0.035), and restricting the clinical model to variables known at diagnosis did not change this (Δ = +0.008; −0.024 to +0.043). Against a null distribution of 1000 random 20-gene sets drawn from the same candidate pool, the signature exceeded the null internally (*p* = 0.015) but was indistinguishable from it in external validation (*p* = 0.154); 99.2% of random sets reached a concordance index above 0.75 and 82.2% above 0.80 in the external cohort. Discrimination fell markedly within the IDH-wildtype (0.629) and WHO grade IV (0.606) strata, and the risk score correlated at 0.722 with a proliferation metagene despite containing no canonical proliferation gene. The concordance indices themselves—0.801 internally and 0.824 externally for the 17-gene subset available in TCGA, with an integrated calibration index of 0.045 at 36 months—are therefore best read as an illustration of the problem rather than as evidence of clinical utility: they sit squarely inside the range that random gene sets reach in the same data, and they fall in the range routinely presented as successful in the published literature. In the literature survey, 8.7% of the 515 records mentioned calibration, 4.5% decision curve analysis, and only 1.0% any comparison against a clinical reference model; none reported all three. **Conclusions:** A signature developed under a leakage-controlled protocol and well calibrated was nevertheless indistinguishable from the appropriate references on two of the three criteria we propose. Most of its discrimination rests on the IDH and grade axis that a broad range of prognostic gene sets can capture. Concordance indices reported in the glioma signature literature cannot be interpreted without a clinical reference model and a random-signature null distribution.

## 1. Introduction

Diffuse gliomas are the most common primary malignant tumours of the adult central nervous system, and their prognosis spans a wide range. The 2021 World Health Organization classification elevated isocitrate dehydrogenase (IDH) mutation status and 1p/19q codeletion to diagnostic criteria, placing molecular information at the centre of classification [1]. This shift rests on comprehensive profiling work showing that diffuse gliomas fall into biologically discrete subsets that are not defined by histology alone [2]. Despite standard therapy [3] and this sharpened classification, survival differences within a single molecular subtype remain substantial, sustaining the search for prognostic tools that add information beyond established markers.

The dominant form of this search is the transcriptomic risk signature. The design of published studies is strikingly invariant: a biological theme is chosen, a related gene set is assembled, a risk score is constructed by LASSO or elastic-net-penalised Cox regression, patients are split into two or three groups at the median score, separation is demonstrated with Kaplan–Meier curves, and a nomogram is presented. Immune response, autophagy, ferroptosis, iron metabolism, lactate metabolism, interferon-γ, telomere maintenance, microRNAs and RNA-binding proteins are only some of the themes following this template [4,5,6,7,8,9,10,11,12,13]. The themes appear interchangeable; the methodology does not change.

Behind this productivity lies a statistical phenomenon that has long been recognised but has not adequately reached the glioma literature. Venet and colleagues compared 47 published breast cancer signatures with random gene sets of identical size and showed that twenty-eight were not significantly better than random signatures and eleven were worse than the median random signature; more than 90% of random signatures larger than one hundred genes were significant outcome predictors [14]. Goh and Wong termed this phenomenon random signature superiority and showed that it is driven by overlap with proliferation genes and by signature size, and that it persists beyond breast cancer, being particularly strong in other cancers in a platform-independent manner [15]. The implication is unambiguous: a significant *p*-value is not evidence that a gene set carries disease-specific information. The pitfalls of deriving prognostic classifiers from high-dimensional expression data were in fact set out two decades ago [16], yet they remain widely unaddressed.

How this problem has been handled in lung cancer is instructive. Tang and colleagues evaluated 42 published signatures within a common meta-analytic framework and reported that twenty-five remained prognostics after adjustment for clinical risk factors, while only eighteen performed significantly better than random signatures [17]. What is notable is that both the clinical adjustment and the random-signature comparison were applied within the same study. We found no evaluation of comparable rigour in the glioma literature.

A second problem concerns the scope of evaluation metrics. The prognostic modelling literature has long emphasised that discrimination and calibration are distinct properties and that calibration is mandatory whenever absolute risk is communicated [18]; current reporting guidance makes this an explicit requirement [19]. Methods for quantifying calibration of survival models from a smoothed curve are also well established [20]. Nevertheless, the concordance index is usually the only quantity reported in glioma signature studies. Rather than assume the size of this gap, we chose to measure it.

A third problem is methodological. In high-dimensional biological data, information leakage between training and test sets inflates reported performance beyond what can be observed under real application conditions; joint preprocessing and joint feature selection are among the most common forms of such leakage [21]. In a signature study, allowing the validation cohort to contribute to scaling statistics or to penalty selection is precisely this type of leakage, and it usually remains invisible.

In the present work we pursued three objectives together. First, we confined every model-building operation—from imputation to penalty selection—to the development cohort and locked the final model together with its coefficients, scaling statistics and baseline hazard, so that the internal validation cohort remained genuinely independent. Second, we tested the signature against a clinical reference model, computing the paired difference within the same bootstrap resamples, and assessed calibration from a smoothed curve rather than from stratum averages. Third, we quantified the reporting practice of the field at abstract level across a corpus of 515 records. Our aim is not to propose another signature but to establish the conditions under which a signature carries value.

## 2. Materials and Methods

### 2.1. Cohorts

**Figure 1:**
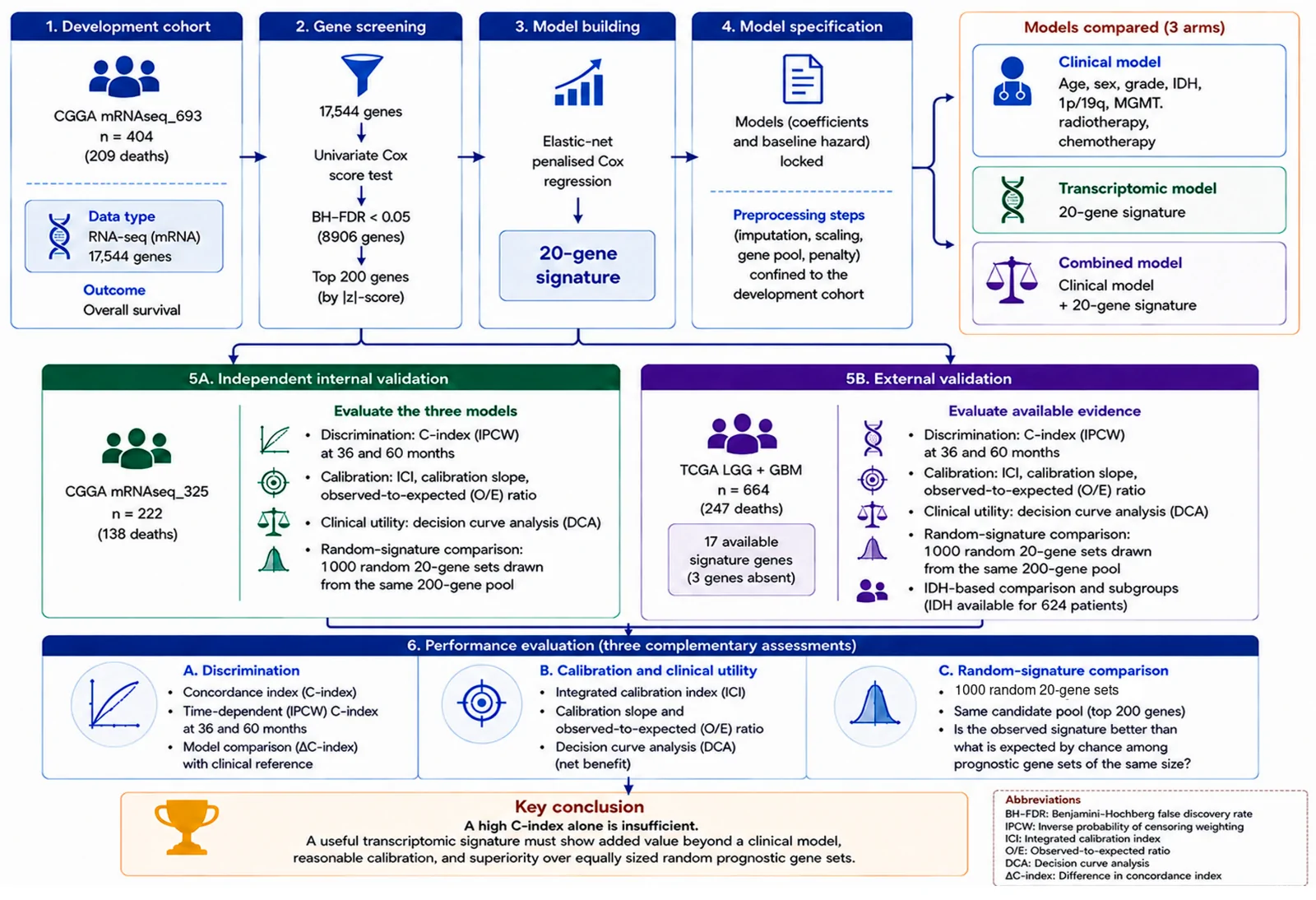
Study design. All model-building operations were confined to the development cohort, and the final models were locked together with their coefficients and baseline hazard. All three arms were fitted with the same elastic-net procedure in the development cohort; the figure highlights only the twenty-gene signature, which is the output of the transcriptomic arm. Abbreviations: BH-FDR, Benjamini–Hochberg false discovery rate; IPCW, inverse probability of censoring weighting; ICI, integrated calibration index; O/E, observed-to-expected ratio; DCA, decision curve analysis.

The study design is summarised in Figure 1. Two independent RNA sequencing cohorts of the Chinese Glioma Genome Atlas (CGGA) were used [22]. Only primary tumours were included; recurrent and secondary cases were excluded. Patients with missing overall survival time or event status were excluded. The mRNAseq_693 cohort (*n* = 404) served as the development set and the mRNAseq_325 cohort (*n* = 222) as an independent internal validation set. For external validation, the lower-grade glioma and glioblastoma studies of the TCGA Pan-Cancer Atlas were combined (*n* = 664), with survival endpoints taken from the curated clinical data resource [23]. The endpoint in all cohorts was overall survival.

### 2.2. Preprocessing and Harmonisation

Expression matrices of the two CGGA cohorts were merged over common genes and log_2_(x + 1) transformed. Genes with a median below 0.5 or a variance below 0.1 were removed, leaving 17,544 genes. TCGA expression data were transformed identically. Scaling statistics of the development cohort were applied to the CGGA-325 cohort, which originates from the same laboratory and platform. Because of platform and population differences, harmonisation in TCGA was performed by within-cohort z-scoring; model coefficients were never re-estimated.

For signature genes unavailable in a target cohort the prespecified rule is as follows: the missing gene is dropped from the risk score, the coefficients of the remaining genes are used unchanged, and no coefficient is re-estimated. No model coefficient was re-estimated using the external cohort; external validation therefore preserved the locked coefficients for all available predictors. The seventeen-gene score is not mathematically identical to the original twenty-gene model; therefore, the effect of this prespecified rule was quantified by re-evaluating the same gene subset in the internal validation cohort.

### 2.3. Candidate Gene Screening

For each gene, the vectorised Cox score-test statistic at β = 0 was computed; this approach yields results for more than seventeen thousand genes within seconds without model fitting. Screening was performed exclusively in the development cohort. Multiplicity was addressed by the Benjamini–Hochberg false discovery rate procedure [24]. At a false discovery rate threshold of 0.05, 8906 genes met the criterion; the top 200 genes ranked by absolute z value formed the modelling candidate pool.

The pool size was fixed at 200 before any validation cohort was examined, on three grounds. First, the development cohort contains 209 events; a pool of 200 candidates therefore corresponds to approximately one event per candidate variable, which is already at the limit of what penalised regression can be asked to absorb, and a substantially larger pool would place selection further into a regime where the choice of genes is governed by sampling noise. Second, the full set of 8906 genes meeting false discovery rate control is not an informative sampling frame in this transcriptome: roughly half of all genes are prognostic on their own (Section 3.3), so that set is closer to a description of the glioma transcriptome than to a shortlist of candidates. Third, and most importantly, the choice does not operate in the signature’s favour. The pool size was varied from 100 to 500 both for discrimination (Supplementary Table S2) and for the random-signature null test (Section 3.9 and Supplementary Table S5); the primary conclusion is unchanged, and the 200-gene pool is in fact the frame in which the signature performs best relative to its own null.

### 2.4. Model Development and Locking

An elastic-net penalised Cox model was fitted along a regularisation path computed by cyclical coordinate descent [25]. The mixing parameter was fixed at 0.9, and the penalty parameter was selected by five-fold cross-validation within the development cohort. Three arms were compared: clinical variables only (age, sex, grade, IDH, 1p/19q, MGMT, radiotherapy, chemotherapy and their missingness indicators), the 200 candidate genes only, and their combination.

The measures taken against leakage must be stated explicitly because such leakage is easily overlooked in high-dimensional biological data and systematically inflates reported performance [21]. The medians used for imputation, the means and standard deviations used for standardisation, the candidate gene pool and the penalty parameter were all computed from the development cohort alone. The internal validation cohort and the external cohort were used at no stage of model construction. The selected coefficients, scaling statistics and the baseline hazard estimated by the Breslow method were written to a file and locked; all subsequent analyses read from that file.

### 2.5. Evaluation

Discrimination was quantified by Harrell’s concordance index [26], with uncertainty from 2000 patient-level bootstrap resamples. Between-arm differences were computed within the same bootstrap resamples, yielding a direct confidence interval for the difference. Time-dependent discrimination was assessed by landmark AUC at 12, 24, 36 and 60 months. The inverse-probability-of-censoring-weighted concordance index [27] was additionally computed at restriction horizons of 36 and 60 months; at longer horizons the support of the censoring distribution is exhausted and the weights become unstable, so those horizons were not evaluated.

Calibration was assessed from a smoothed curve obtained by fitting a Cox model with a three-knot restricted cubic spline on the complementary log–log transformation of the predicted survival probability. The integrated calibration index is the mean absolute difference between predicted and smoothed observed probabilities; E50 and E90 are the median and ninetieth percentile of the same difference [20,28]. For comparison, the stratum-based approximate measure commonly used in the literature was also computed. The calibration slope and the observed-to-expected ratio were additionally reported. Clinical utility was assessed by decision curve analysis for 36-month mortality [29].

### 2.6. Construction of the Random-Signature Null Distribution

Goh and Wong proposed that the criterion should not be the significance of a signature but its superiority over a null distribution of random gene sets of identical size and recommended restricting the null sample to sets that do not overlap the observed signature [15]. We applied this recommendation to our own signature. The sampling frame is the modelling candidate pool itself: the top 200 screened genes, excluding the twenty genes of our signature, leaving 180. This frame is identical to the pool available to the selection procedure, so the test directly measures whether selection gained anything over a random draw from that pool. From this frame, 1000 random twenty-gene sets were drawn. As a sensitivity analysis the same procedure was repeated with a wider frame of the top 500 screened genes. Each set was subjected to exactly the same procedure as the observed signature: scaled with the statistics of the development cohort, fitted by an L2-penalised Cox model in the development cohort, and transferred unchanged to the internal validation and external cohorts. The null *p*-value was defined as the proportion of random sets attaining a concordance index equal to or greater than the observed one.

It should be noted that this null distribution is more demanding than the construction of Venet and colleagues: random sets are drawn not from the whole transcriptome but from the genes that passed false discovery rate control and were nominated as modelling candidates. The test therefore asks not whether our signature consists of prognostic genes but whether the selection procedure gained anything over a random draw from the same pool. This matching between the sampling frame and the selection pool is not a technical detail but the property that determines what the test can establish; its quantitative consequences are reported in Section 3.9.

### 2.7. Methods for Sensitivity and Mechanistic Analyses

Including radiotherapy and chemotherapy in the clinical reference model may introduce bias in observational cohorts because access to treatment depends on survival time. The clinical arm was therefore rebuilt using only variables known at diagnosis (age, sex, grade, IDH, 1p/19q, and MGMT), and the comparison with the signature was repeated against that model. Because the proportional hazards assumption is violated in the internal validation cohort, restricted mean survival time (RMST), which does not require that assumption, was computed as an additional measure. RMST differences between the risk groups defined by the median of the development cohort were obtained at 36 and 60 months with 1000-resample bootstrap intervals. To assess the extent to which discrimination derives from proliferation-associated gene programmes, a metagene was constructed from the sixteen canonical proliferation genes present in the candidate pool (MKI67, CCNB1, BIRC5, AURKA, RRM2, CDK1, CCNB2, BUB1, KIF20A, UBE2C, TPX2, MCM2, FOXM1, CENPF, ASPM and NUSAP1). The metagene is the mean of expression values scaled with development-cohort statistics. The association of the signature risk score with the metagene, the discrimination of the metagene alone, and the information the signature adds beyond the metagene were evaluated separately.

### 2.8. Quantification of the Field’s Reporting Practice

To assess the extent to which key components of prognostic model evaluation are explicitly reported in the glioma transcriptomic-signature literature, we conducted an abstract-level survey of records indexed in the Web of Science Core Collection. The database was searched on 9 August 2026 using all editions available through the institutional subscription (Editions: All); the eight included indexes and their respective coverage periods are detailed in Supplementary Methods S1. Records were eligible if the Topic field contained either glioma or glioblastoma; at least one of the terms signature or risk score; and at least one of the terms LASSO, elastic net, or nomogram. No restrictions were imposed on publication year, language, or document type. The search yielded 534 records, with no title-level duplicates identified. Nineteen records were excluded because neither glioma nor glioblastoma appeared in the title, abstract, or keywords, leaving 515 eligible records published between 2014 and 2026. All retained records represented journal publications. However, because the document-type field was not included in the exported dataset, the distribution of specific publication types could not be independently verified. Accordingly, the term record rather than article is used throughout this analysis. One record lacked an abstract and was therefore screened on the basis of its title and keywords alone. The complete search strategy, eligibility criteria, prespecified regular-expression patterns, and record-selection process are provided in Supplementary Methods S1. For each eligible record, the title, abstract, and keywords were systematically screened using prespecified regular-expression patterns to identify explicit reporting of calibration, decision curve analysis, concordance index, Brier score, and comparison with a clinical reference model. The pattern used to identify clinical-model comparisons was intentionally broad and encompassed terminology referring to comparison with clinical variables, added or incremental predictive value, and superiority over a clinical or clinicopathological reference model. Because this survey was restricted to abstract-level reporting, it was designed to capture only those evaluation practices explicitly mentioned in the title, abstract, or keywords. Analyses that may have been performed and reported exclusively in the full text were therefore not counted. Consequently, the observed proportions should be interpreted as conservative, lower-bound estimates of the frequency with which these methodological and performance-evaluation practices are explicitly reported in the glioma signature literature.

### 2.9. Reproducibility

All computations were carried out in Python 3.12. The random seed was fixed at 42 at every stochastic step. The pipeline produced byte-identical checksums for all output files across two independent runs; a verification script that compares output signatures against a stored reference is included in the code repository.

## 3. Results

### 3.1. Cohort Characteristics

Characteristics of the three cohorts are given in Table 1. The development and internal validation cohorts are comparable in age and IDH mutation frequency; the internal validation cohort has a higher event rate (62.2% versus 51.7%) and a shorter median survival (44.7 versus 63.5 months). The external cohort has a higher proportion of IDH-mutant tumours (63.0%) and a markedly shorter median follow-up (29.2 months).

### 3.2. Reporting Practice of the Field

Of the 515 glioma signature records, 77.7% reported LASSO or an equivalent penalised selection method, 67.4% a risk score and 51.3% a nomogram; 47.0% used CGGA cohorts for validation. By contrast, calibration was mentioned in 8.7% and decision curve analysis or net benefit in 4.5%. No record mentioned the Brier score. Comparison against a clinical reference model appeared in 1.0%, and four of those five records were radiomics rather than signature studies. No record reported all three of discrimination, calibration and clinical comparison (Table 2).

Two features of this survey require comment. The first is the criterion for comparison with a clinical reference model, which was deliberately broad: a record was counted if its title, abstract or keywords referred in any form to comparison with clinical variables, to added or incremental predictive value over clinical or clinicopathological information, or to superiority over a clinical reference model. No requirement was imposed that the comparison be quantitative, paired, or fitted in the authors’ own data. The 1.0% figure is therefore an upper bound under a permissive definition rather than a strict count, which strengthens rather than weakens the observation.

The second is that 47.0% of records used CGGA cohorts for validation. On its own this is a neutral observation about data availability: CGGA is one of the few large glioma cohorts with paired RNA sequencing and survival data in the public domain. It acquires interpretive weight when read together with the present results. Nearly half the field validates in the same cohort family in which our own signature crossed the null threshold (*p* = 0.015) while failing to do so in an independent external cohort (*p* = 0.154). Concentration of validation in a single cohort family means that the discrimination reported across the literature is repeatedly estimated under correlated conditions, so agreement between studies cannot be taken as evidence of transportability. This is a reason to require external validation in a different cohort family, not a criticism of any individual study.

The most striking inconsistency in this picture is between nomograms and calibration. A nomogram is a device that communicates absolute risk to a patient; yet 264 records presented a nomogram against 45 that mentioned calibration. The abstract-level nature of the analysis may depress this proportion, but the magnitude of the difference cannot be explained by measurement alone.

### 3.3. Screening and Signature

**Figure 2:**
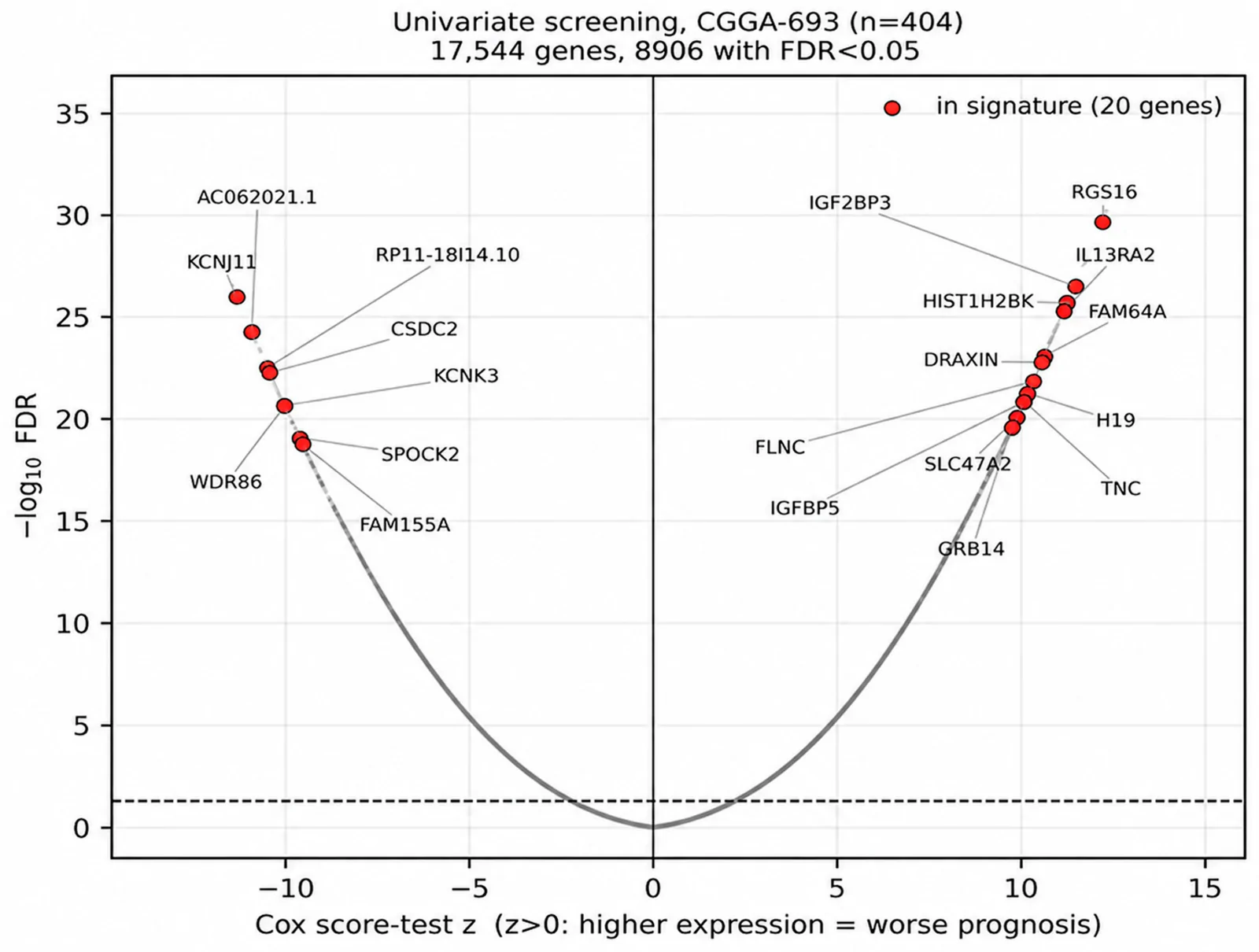
Univariate screening in the development cohort. The horizontal axis shows the Cox score-test statistic and the vertical axis the negative logarithm of the false discovery rate. Red points mark the 20 genes retained in the final signature. The dashed horizontal line indicates the statistical significance threshold at FDR = 0.05 [−log_10_(0.05) ≈ 1.30]; genes above this line were considered statistically significant after false discovery rate correction.

Of the 17,544 genes screened in the development cohort, 8906 met the false discovery rate threshold of 0.05 (Figure 2). This proportion is important because it shows how pervasive survival-associated signal is in the glioma transcriptome: roughly half of all genes are prognostic on their own. The figure is consistent with the observation that at least half of the breast cancer transcriptome correlates with a proliferation metagene [14,15] and explains why a seemingly successful signature can be produced from an arbitrary gene set.

The elastic-net model selected 20 genes from the 200 candidates. Risk-increasing genes included GRB14, RGS16, FAM64A, H19, HIST1H2BK, IGFBP5, IL13RA2, TNC and IGF2BP3; protective genes included SPOCK2, KCNK3, KCNJ11, WDR86, FAM155A and CSDC2. The full coefficient list is given in Supplementary Table S1.

### 3.4. Discrimination

**Figure 3:**
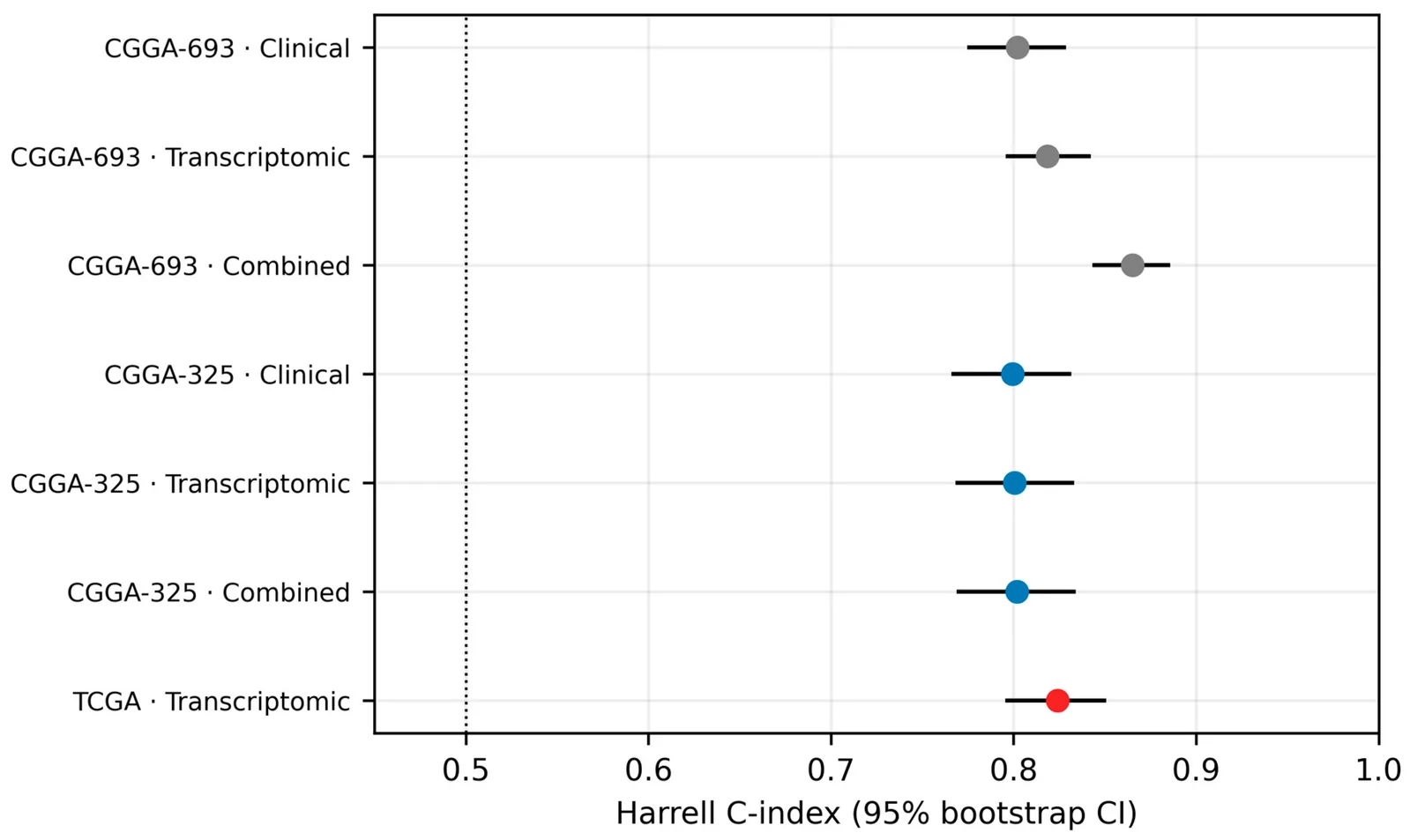
Concordance indices of the three modelling arms with 95% bootstrap confidence intervals. Grey points represent the development cohort (CGGA-693), blue points represent the independent internal validation cohort (CGGA-325), and the red point represents the external validation cohort (TCGA). Horizontal error bars indicate 95% bootstrap confidence intervals. The dotted vertical line at a concordance index of 0.50 indicates discrimination no better than chance.

In the development cohort the combined model gave the highest concordance index, as expected (0.865). In the independent internal validation cohort the three arms became indistinguishable: clinical model 0.800 (95% CI 0.767–0.831), transcriptomic signature 0.801 (0.769–0.832), combined model 0.802 (0.770–0.833) (Figure 3). The paired bootstrap differences include zero: combined versus clinical Δ = +0.003 (−0.023 to +0.029), signature versus clinical Δ = +0.001 (−0.032 to +0.035). The advantage of 0.065 observed in the development cohort did not transfer to the independent cohort.

Three of the twenty signature genes—AC062021.1, DRAXIN and RP11-18I14.10—are absent from the TCGA expression matrix; two are non-coding RNAs designated by clone identifiers with no counterpart in the HUGO symbol-based representation of the TCGA Pan-Cancer dataset. Under the prespecified rule these three genes were dropped from the risk score, and the coefficients of the remaining seventeen were used unchanged. The cost of this rule was measured in the internal validation cohort: the same seventeen-gene subset yields a concordance index of 0.800, practically indistinguishable from the 0.801 of the full twenty-gene signature. In the external validation cohort the seventeen-gene model achieved 0.824 (0.797–0.850); discrimination was preserved despite a different country, a different sequencing platform and a different follow-up structure.

**Figure 4:**
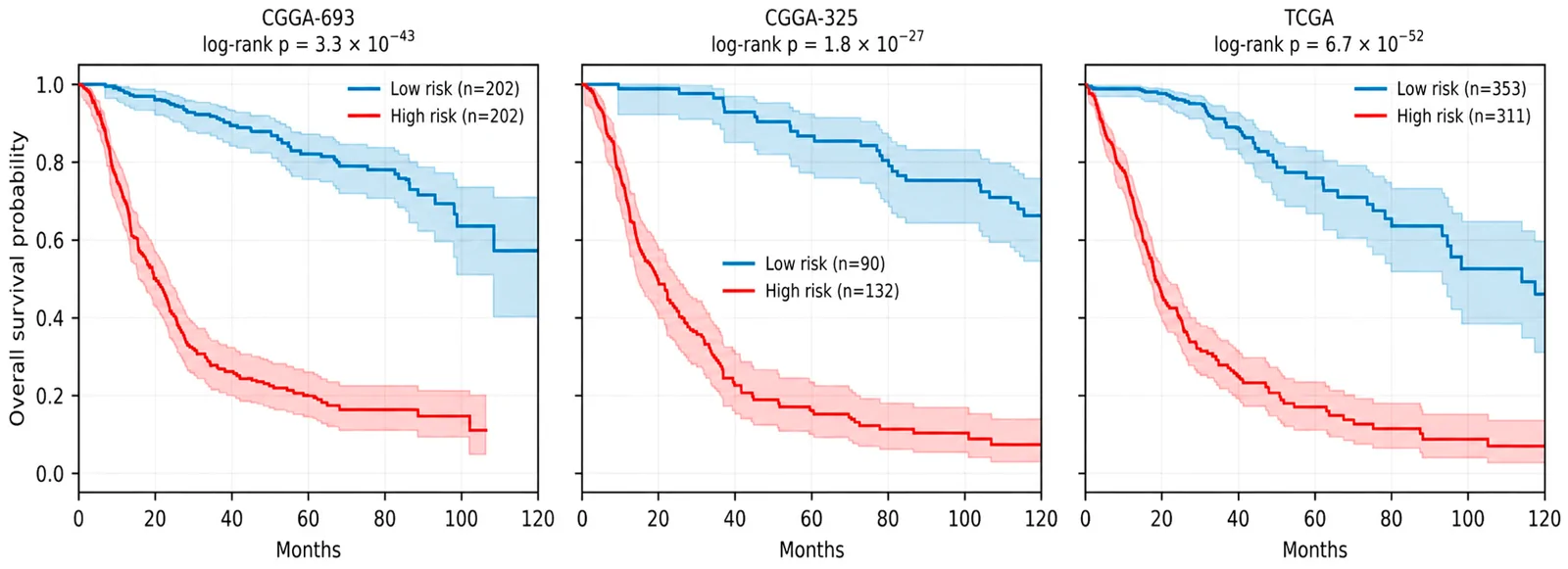
Kaplan–Meier curves for risk groups defined by the median of the development cohort. The threshold was not re-derived in the validation cohorts.

When the risk threshold was fixed at the median of the development cohort and applied unchanged, median survival was not reached in the low-risk group of the internal validation cohort and was 19.8 months in the high-risk group (log-rank *p* = 1.8 × 10^−27^). The corresponding values in the external cohort were 114.1 and 18.4 months (*p* = 6.7 × 10^−52^) (Figure 4).

### 3.5. Independence from Established Markers

When the signature was added to a multivariable Cox model containing grade, IDH, 1p/19q and MGMT status in the internal validation cohort, model fit improved significantly (χ^2^ = 29.5; *p* = 5.6 × 10^−8^); the hazard ratio of the signature was 2.29 (*p* < 0.001), exceeding the coefficients of all markers other than grade. The concordance index rose from 0.792 to 0.807.

Only IDH subtype information was available in the external cohort. In this subsample restricted to the 624 patients with a reported IDH subtype, the signature alone achieved a concordance index of 0.829 and IDH status alone 0.790; the difference from the 0.824 reported for the full cohort arises solely from the subsample (SUBTYPE was missing for 40 patients). When modelled jointly, the likelihood ratio test (χ^2^ = 42.9; *p* = 5.7 × 10^−11^) demonstrated the independent contribution of the signature. Within the IDH-mutant stratum, median survival differed between risk groups (114.1 versus 63.5 months; *p* = 2.4 × 10^−5^), and within the IDH-wildtype stratum the contrast was between 15.0 months and a median that was not reached (*p* = 2.5 × 10^−2^).

### 3.6. Calibration

At 36 months the integrated calibration index was 0.053 in the development cohort, 0.067 in the internal validation cohort and 0.045 in the external cohort (Table 3). The calibration slope in the external cohort was 1.18 and the observed-to-expected ratio 0.92. These values indicate that absolute risk transferred with reasonable accuracy even though the baseline hazard was taken from the development cohort and never re-estimated in the target cohort.

The extent to which the choice of measure alters the result deserves attention. The mean absolute deviation computed over four strata was 0.064 at 36 months in the external cohort, whereas the integrated calibration index computed from the smoothed curve was 0.045. The difference has two sources. The stratum-based measure is computed from only four points and therefore assigns equal weight to extreme strata where patient density is low, whereas the integrated calibration index weights the difference by the distribution of predicted risk. In addition, averaging within a stratum allows deviations of opposite sign to cancel and cannot capture the shape of the curve. The two measures should not be used interchangeably.

**Figure 5:**
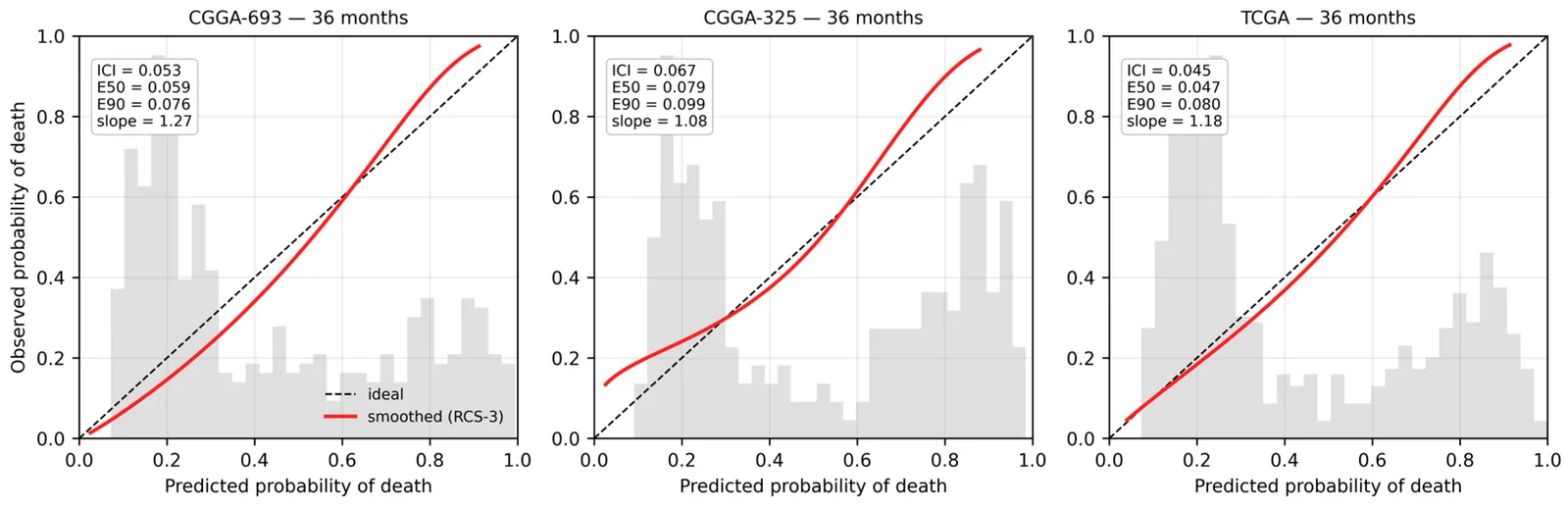
Calibration curves at 36 months. The grey histogram shows the distribution of predicted risk.

A recalibration in which the slope and baseline hazard were re-estimated in the target cohort, leaving model coefficients untouched, was attempted in the external cohort. To avoid evaluating the recalibrated model on the data used to recalibrate it, the external cohort was randomly split in half 50 times; the slope and baseline hazard were re-estimated in one half and the integrated calibration index computed in the other, and the values below are means across the 50 splits. The index moved from 0.027 to 0.046 at 12 months, from 0.048 to 0.046 at 24 months, from 0.058 to 0.047 at 36 months and from 0.066 to 0.053 at 60 months: an improvement at the three longer horizons and a clear deterioration at 12 months. These half-sample values are not directly comparable with those in Table 3, which are computed in the full external cohort (0.045 at 36 months); the integrated calibration index is upward biased in smaller samples, which accounts for the difference (Figure 5). Since recalibration produced no consistent improvement over calibration that was already adequate, it was not adopted.

### 3.7. Clinical Utility

**Figure 6:**
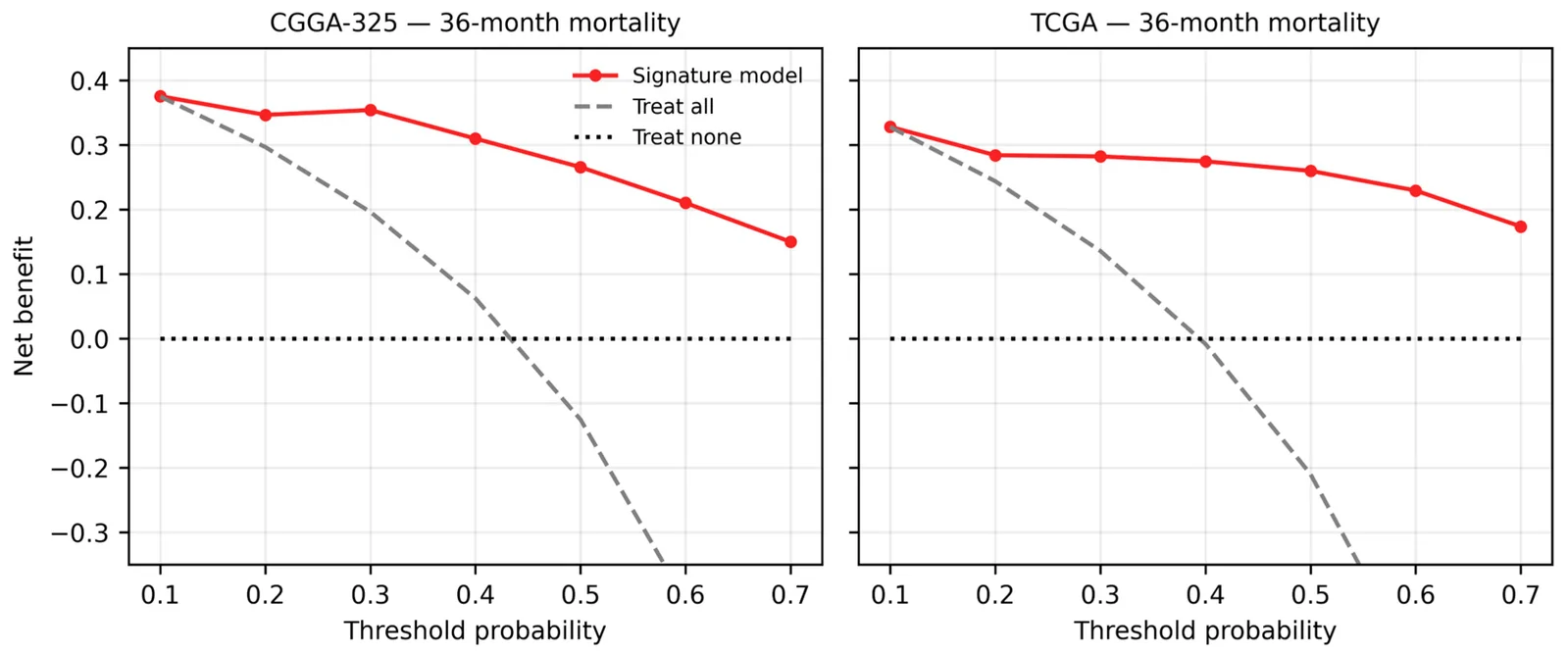
Decision curve analysis for 36-month mortality.

In decision curve analysis for 36-month mortality, the transcriptomic signature model provided positive net benefit across a wide range of thresholds in both validation cohorts. In the external cohort, net benefit at a threshold of 0.30 was 0.282 against 0.136 for the strategy of treating all patients as high risk; beyond a threshold of 0.40 the latter strategy became negative while the model remained positive even at 0.70 (Figure 6).

### 3.8. Robustness and Sensitivity Analyses

The influence of modelling choices is collected in Supplementary Table S2. Varying the candidate pool size from 100 to 500 changed the concordance index in the independent cohort between 0.775 and 0.801; varying the mixing parameter between 0.7 and 1.0 left it at 0.801, falling to 0.764 only at 0.5. The values used in the primary analysis (200 candidates, mixing parameter 0.9) were fixed at the outset, before the validation cohort was seen. Restricting the analysis to the 194 patients with complete clinical data gave 0.799. Changing the harmonisation method in the external cohort barely altered the result: within-cohort z-scoring 0.824, the development cohort scaler 0.822, rank transformation 0.821.

The magnitude of the effect that leakage control has on reported performance can be quantified directly from the history of this analysis. In our own first pass we estimated the scaling statistics and the penalty parameter across the whole CGGA sample rather than within the development cohort alone. Recognising this as joint preprocessing and joint feature selection leakage [21], we corrected the protocol and relocked the model. The concordance index in the independent internal validation cohort fell from 0.827 to 0.801. The inflation is therefore approximately 0.026 in concordance—a difference that would ordinarily be presented as a meaningful performance advantage over a competing model, produced here by nothing more than two preprocessing steps that are routinely applied to pooled data and rarely reported. Two considerations make this figure worth stating explicitly. It is measured within a single protocol, so it is not confounded by differences in cohort, endpoint or model class; and it is a lower bound because the leakage in question involved neither the candidate pool nor the outcome, which are the forms of leakage capable of producing far larger distortions.

Optimism in the development cohort was estimated by bootstrap. The apparent concordance index was 0.819, optimism 0.019 and the corrected value 0.800, which corresponds almost exactly to the 0.801 observed in the independent cohort. This agreement is direct evidence that the leakage-controlled protocol worked as intended.

Time-dependent discrimination was also assessed by the inverse-probability-of-censoring-weighted concordance index; at a sixty-month horizon it was 0.812 in the internal validation cohort and 0.826 in the external cohort, consistent with the Harrell index. Testing the proportional hazards assumption with Schoenfeld residuals showed that it holds in the development cohort (*p* = 0.86), is borderline in the external cohort (*p* = 0.066) and is violated in the internal validation cohort (*p* = 0.027); the effect of the risk score attenuates over time. Concordance indices and calibration estimates at long horizons should therefore be interpreted with corresponding caution. The limitation is most acute at the 60-month horizon in the external cohort. Median follow-up there is 29.2 months, and by 60 months only 68 of 664 patients (10.2%) remain at risk, with 218 of the 247 observed events having already occurred. The 60-month calibration and restricted mean survival time estimates in this cohort therefore rest on a small and increasingly selected remainder of the sample, and the accompanying confidence intervals understate that uncertainty because they do not account for the instability of the tail of the Kaplan–Meier estimate. These values are reported for completeness and should not be read as evidence about five-year performance; the 24- and 36-month horizons, where 264 and 164 patients respectively remain at risk, are the ones on which our conclusions rest.

Subgroup analysis yields one of the most informative results of the study. In the internal validation cohort the concordance index falls from 0.794 in the IDH-mutant stratum to 0.629 in the IDH-wildtype stratum and from 0.707 and 0.759 in WHO grades II and III to 0.606 in grade IV. The corresponding values in the external cohort are 0.683 for IDH-mutant and 0.563 for IDH-wildtype. A substantial part of the overall discrimination therefore arises from separation between strata, that is, along the IDH and grade axis.

### 3.9. Results of the Random-Signature Null Distribution Analysis

**Figure 7:**
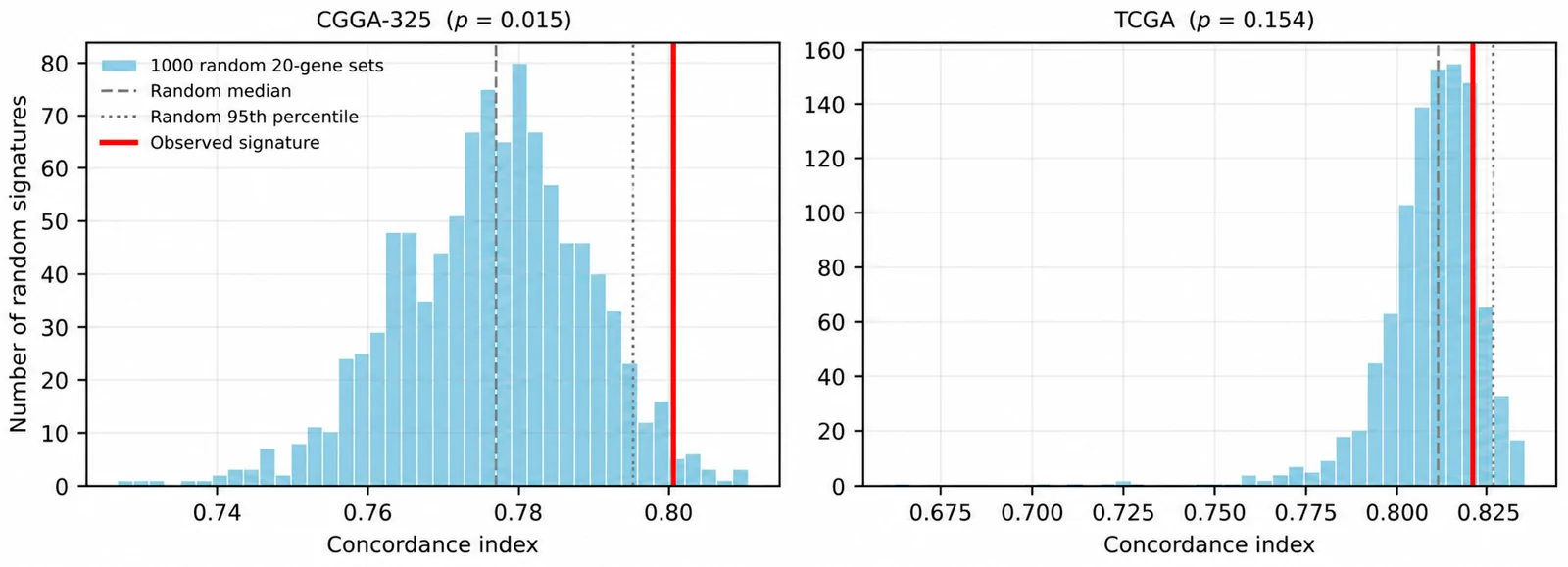
Distribution of concordance indices for 1000 random twenty-gene sets drawn from the candidate pool. The red line marks the observed signature, the dashed line the random median and the dotted line the ninety-fifth percentile.

The distribution of concordance indices produced by 1000 random twenty-gene sets drawn from the candidate pool is shown in Figure 7. In the internal validation cohort the random sets had a median of 0.777 (interquartile range 0.768–0.785) and a ninety-fifth percentile of 0.795; the observed value of 0.801 was matched or exceeded by 1.5% of random sets (*p* = 0.015). In the external cohort the random median was 0.811 and the ninety-fifth percentile 0.826, and the observed value of 0.821 was matched or exceeded by 15.4% of random sets (*p* = 0.154). The signature therefore exceeds the null distribution in the internal validation cohort but is indistinguishable from it in the external cohort.

The dependence of this test on the sampling frame was examined in two ways (Supplementary Table S5). First, the entire procedure was repeated within alternative candidate pools: for pools of 100 and 500 genes the signature was rebuilt from scratch under the identical protocol, and the null was drawn from that same pool with sets matched to the size of the resulting signature. With the 100-gene pool the procedure selected 48 genes and the resulting score fell below the median of its own null in both validation cohorts (*p* = 1.000 and *p* = 0.983); for the 500-gene pool it selected 58 genes and again failed the test (*p* = 0.185 and *p* = 0.748). The primary 200-gene pool is thus the frame in which the signature performs best relative to random draws, and even there it does not exceed the null externally. The negative external result is consequently not an artefact of the pool size, and the pool size chosen cannot be said to have favoured the signature.

Second, the frame effect was isolated by holding the signature and the random set size fixed at twenty genes and varying only the frame from which the random sets were drawn. As the frame widens from the top 100 to the top 200 and then the top 500 screened genes, the median of the null distribution in the external cohort falls from 0.820 to 0.811 and then to 0.808, and the null *p*-value moves correspondingly from 0.473 to 0.154 and then to 0.129. The narrower the frame, the more demanding the test because the random sets are then drawn from genes that are themselves strongly prognostic. This has a direct implication for how such tests should be constructed. A null distribution drawn from the whole transcriptome would consist largely of sets with little prognostic content, would sit far below the observed value, and would return a significant *p*-value for essentially any signature selected from a screened pool. The null must therefore be drawn from the same candidate pool that was available to the selection procedure; otherwise the test measures the success of the screening step, which is not in question, rather than the success of selection, which is.

The location of these distributions is more striking than the test result itself. Of the random twenty-gene sets, 97.6% in the internal validation cohort and 99.2% in the external cohort achieved a concordance index above 0.75, and 82.2% exceeded 0.80 in the external cohort. Values presented as evidence of success in published glioma signatures typically fall in this range. A concordance index in this range cannot therefore, on its own, serve as evidence that information specific to the selected gene set is being carried.

### 3.10. Results of Sensitivity and Mechanistic Analyses

When the clinical reference model was rebuilt using only variables known at diagnosis, its concordance index fell from 0.800 to 0.792 and the paired difference against the signature rose from +0.001 to +0.008; the confidence interval nevertheless continued to include zero (95% CI −0.024 to +0.043). The principal result is therefore unaffected by the removal of treatment variables from the model.

Restricted mean survival time was computed as an additional measure because it does not require the proportional hazards assumption. The difference between the risk groups defined by the median of the development cohort was 14.2 months (95% CI 12.0–16.3) at 36 months and 31.3 months (27.4–34.9) at 60 months in the internal validation cohort, and 14.2 months (12.6–15.7) and 28.8 months (25.6–31.6) respectively in the external cohort. Separation between risk groups is therefore preserved under an assumption-free measure.

The proliferation metagene analysis points directly to the source of discrimination. The signature risk score is strongly associated with the metagene (Spearman rho = 0.722; *p* = 5.4 × 10^−37^), even though none of the twenty signature genes is a canonical proliferation gene. The metagene alone yields a concordance index of 0.723; the signature exceeds this at 0.801 and adds significant information beyond it (χ^2^ = 67.3; *p* = 2.3 × 10^−16^). Of the 500 genes in the candidate pool, 46.2% show an absolute correlation greater than 0.5 with the metagene.

The loss of the three signature genes unavailable in TCGA is not selective. The TCGA expression matrix contains 85.0% of the signature genes, 91.5% of the top 200 candidates and 89.8% of the top 500 screened genes, so the loss within the signature is close to the base rate. Of the 51 genes missing from the top 500, 35% are non-coding RNAs designated by clone or LINC identifiers with no counterpart in the HUGO symbol-based representation of the TCGA Pan-Cancer dataset. The absence is therefore a property of the annotation, not of the biology. The consequence for discrimination deserves a more careful reading than it is usually given. Evaluated in the internal validation cohort, the 17-gene subset attains a concordance index of 0.800 against 0.801 for the full 20-gene score: removing three of twenty predictors costs essentially nothing. It is tempting to present this as robustness, and in the narrow operational sense it is—the prespecified gene-dropping rule does not degrade the score when it is applied. But robustness of this kind is difficult to separate from redundancy, and the remaining evidence points to redundancy. The risk score correlates at 0.722 with a proliferation metagene that shares no gene with the signature, 46.2% of the candidate pool correlates above 0.5 in absolute value with that same metagene, and random 20-gene sets drawn from the pool reach a concordance index above 0.80 in 82.2% of draws in the external cohort. A score whose performance is insensitive to the identity of its constituent genes is behaving as a summary of a broad transcriptional axis rather than as a specific gene set. On this reading insensitivity to gene loss is not an independent strength of the signature but a further expression of the same finding that the null distribution reports, and it is consistent with the interpretation that the discrimination is carried by the IDH-grade-proliferation axis rather than by the twenty genes as such.

## 4. Discussion

### 4.1. Principal Finding: Independence and Added Value Are Not the Same

The central finding of this study is a dissociation. The 20-gene signature carries prognostic information that is statistically independent of established markers; yet in an independent cohort with complete clinical annotation it makes no measurable contribution to overall discrimination. The two statements appear contradictory but are not. The likelihood ratio test shows that the signature has an effect on the hazard that is not explained by the other variables; the concordance index measures the ordering of patient pairs, and for pairs that the available variables already order correctly, additional information need not translate into a visible gain.

This distinction has a direct consequence for the field. Significance of a signature in a multivariable hazard model does not imply that it will contribute to clinical decision-making. In our own data the advantage of the combined model over the clinical model was 0.065 in the development cohort and did not transfer at all to the independent cohort. Without a reference model such an advantage is reported as a genuine gain and cannot subsequently be corrected.

### 4.2. Where the Signature Is Actually Useful

The value of the signature emerges elsewhere. Grade, 1p/19q and MGMT status were unavailable in the external validation cohort; there the signature alone achieved a concordance index of 0.824 and outperformed IDH status, the only available marker. The potential value of the signature therefore lies not in being a layer added to clinical information but in the possibility of serving as a prognostic substitute where complete clinical and molecular annotation is unavailable; this possibility would require prospective testing in such settings. This corresponds to the real situation of centres with limited access to retrospective data sources and molecular pathology. The signature also retained discriminative ability within both IDH strata, indicating that it captures part of the within-stratum heterogeneity.

### 4.3. Applying the Proposed Criterion to Our Own Signature

When the third criterion we propose is applied to our own signature, the signature exceeds the null distribution of random twenty-gene sets drawn from the same candidate pool in the internal validation cohort but is indistinguishable from it in the external cohort. We interpret this not as a weakness peculiar to our signature but as an indication of a measurement problem that applies across the field; we found no instance of the same test being applied to any published glioma signature, and there is no reason to expect a different outcome.

The location of the null distribution changes how discrimination values should be read. Almost all random twenty-gene sets drawn from the prognostic gene pool exceed the threshold of 0.75 that is presented as success in the literature. This is the glioma counterpart of the pattern reported by Venet and colleagues in breast cancer [14] and is consistent with our own finding that half of all genes are prognostic on their own. As Goh and Wong emphasised, rejection of the null hypothesis is insufficient under these conditions [15].

The proliferation metagene analysis demonstrates the mechanism directly. Although our signature contains no canonical proliferation gene, its risk score is associated with the metagene at 0.722, and almost half of the genes in the candidate pool correlate strongly with it. This is the glioma counterpart of the pattern reported by Venet and colleagues in breast cancer and confirmed by Goh and Wong across several tumour types [14,15,30,31]: because proliferation-associated gene programmes are distributed across much of the transcriptome, a set drawn at random from prognostic genes also carries this information. That the signature adds significant information beyond the metagene (χ^2^ = 67.3) shows that this information is not exhausted by proliferation, but it explains why the margin over random sets remains small. Subgroup analysis points to the same conclusion. Discrimination weakens markedly within the IDH-wildtype and grade IV strata. Most of the overall performance therefore derives from ordering patients along the molecular and histological axis that already determines prognosis to a large extent. Because a broad range of prognostic gene sets can capture that axis, the high performance of random sets and the failure of our signature to separate from them are two aspects of the same phenomenon. The same pattern was reported in the contemporaneous study, where the concordance index also fell to 0.59 in the IDH-wildtype stratum [32].

This finding does not invalidate the other results of the study. The signature adds significant information to the multivariable model, is well calibrated, and outperforms IDH status in a cohort without clinical annotation. None of this, however, means that the signature is better than a random selection of prognostic genes. Both statements are true simultaneously, and the fact that the field usually reports only the first is the source of the problem.

### 4.4. Comparison with Contemporaneous Work

While the present work was being prepared, a study using the same two cohort families and motivated by a similar rationale was published. D’Costa and colleagues addressed reproducibility and overfitting in the glioma signature literature by means of stability selection; their 20-gene signature, developed in the TCGA cohort (n = 694) and validated in the CGGA cohort (n = 657), achieved a concordance index of 0.7392 [32], outperforming most of fourteen published benchmark models.

The differences between that study and ours sharpen rather than eliminate our contribution. First and most importantly, that study did not construct a clinical reference model; the authors state this explicitly in their limitations, noting that direct comparison with clinical models based on IDH and 1p/19q was beyond the scope of their analysis and was left to future work. The clinical model value appearing in their comparison table was taken from the literature rather than computed in their own data. That comparison is the primary question of the present study.

Second, calibration is handled differently. That study assessed calibration by Brier scores and by points representing the means of three risk groups; in the external validation cohort the reported Brier scores were 0.4072 at one year, 0.2382 at three years and 0.1932 at five years, with only the five-year value highlighted in the abstract. A value of 0.41 at one year indicates that short-horizon absolute risk estimates were not reliable in the external cohort. A three-point calibration assessment does not provide the resolution of an index computed from a smoothed curve; in our data we showed that even a four-stratum approach differs from the true index by roughly forty percent.

Third, the two studies differ in leakage control. In that study feature selection used stability selection [33] with mutual information against tumour class in addition to survival; because tumour class encodes grade, this choice may weaken the claim that the signature is independent of grade. In our protocol selection was based on survival alone and was confined to the development cohort. The magnitude of the inflation that leakage of this kind can produce is quantified in Section 3.8: an equivalent error in our own first pass, recognised as joint preprocessing and joint feature selection leakage [21], reduced the internal validation concordance index by 0.026 once corrected.

Finally, the concordance indices of the two studies should not be compared directly: cohort definitions differ (primary tumours only in our case), the direction of development and validation differs (CGGA→TCGA here and TCGA→CGGA there), and event rates differ. Our higher values partially arise from all grades being represented within a single cohort.

### 4.5. What the Reporting Survey Implies

Across 515 records, not one reported discrimination, calibration and a clinical reference comparison together. This reflects an established template in the field rather than the failure of individual studies. Reporting recommendations for tumour marker studies were defined two decades ago [34], and current guidance for prognostic model reporting makes calibration an explicit requirement [19]; the gap nevertheless persists.

Why this gap matters becomes clearer alongside the second finding of our screening. Of 17,544 genes, 8906 were prognostic on their own. In a transcriptome where signal is this pervasive, producing an apparently significant risk score from a gene set assembled around any biological theme is close to inevitable. The phenomenon demonstrated by Venet and colleagues in breast cancer [14] and by Goh and Wong across multiple tumours [15] has the biological substrate to hold in glioma as well. In such a field significance alone cannot serve as evidence of information; the criterion must be superiority over an appropriate reference.

We therefore propose three requirements as a minimum standard for glioma signature studies: comparison against a clinical reference model containing grade and molecular markers, with a paired measure of uncertainty; calibration from a smoothed curve for any model that communicates absolute risk; and testing against a null distribution of random gene sets of identical size [15]. We applied all three in the present study and report the results as they stand, including those unfavourable to our own signature.

### 4.6. Biological Coherence of the Signature Genes

Although the signature was selected by statistical criteria, the directions of effect are largely consistent with established biology. Direct evidence in glioma exists for six of the risk-increasing genes. RGS16 expression increases with glioma grade, concentrates in mesenchymal and IDH-wildtype tumours, and has been reported as an independent indicator of poor prognosis [35]. High PIMREG (FAM64A) expression is associated with grade and with poor survival even in lower-grade gliomas [36]. The long non-coding RNA H19 is overexpressed in glioma tissue and promotes proliferation and invasion through the Wnt/β-catenin pathway [37]. IL13RA2 is overexpressed in malignant gliomas, concentrates in IDH-wildtype and TERT promoter-mutant glioblastomas, and is associated with poor survival [38]. Tenascin-C promotes invasion and angiogenesis through extracellular matrix remodelling and the behaviour of tumour-associated microglia and macrophages [39]. IGF2BP3, an m^6^A reader, enhances proliferation through cell-cycle regulators [40]. In all six cases the direction reported in the literature matches the sign of the coefficient in our model.

Two genes require separate comment. For GRB14, the variable with the largest coefficient, we found no published prognostic study in glioma; high expression has been associated with advanced stage and shorter survival in colorectal cancer [41]. SPOCK2, protective in our model, encodes the extracellular matrix proteoglycan testican-2, which has been shown to limit vitronectin–integrin interaction and thereby to dampen matrix-based signalling [42]; the corresponding function in glioma has not been characterised. Both genes are candidates requiring confirmation.

Caution is warranted regarding the evidential weight of this coherence. Proliferation-associated gene programmes have been shown to correlate with a large fraction of the transcriptome and to constitute the principal confounder of signature performance [15,30]. In a field where half of all genes are prognostic on their own, obtaining a list that appears biologically meaningful in retrospect is an expected outcome. Directional agreement supports the conclusion that the signature is not noise, but it is not sufficient for a causal interpretation.

### 4.7. Limitations

First, clinical annotation is not equivalent across the development and validation cohorts. Because grade, 1p/19q and MGMT status are absent in the external cohort, comparison against the clinical reference model was possible only within CGGA. Second, the clinical reference model includes radiotherapy and chemotherapy, which are recorded after diagnosis and may be associated with survival through treatment selection; a sensitivity analysis restricted to variables known at diagnosis is reported and does not alter the conclusion. Third, strong prognostic variables such as performance status and extent of resection are unavailable in all cohorts; our clinical reference model is therefore not the strongest possible one, and the true advantage of a clinical model may be larger than we report. Third, the number of events in the development cohort falls below recommended minimum sample size criteria relative to the number of candidate variables [43]; penalised regression limits but does not eliminate this risk. Fourth, median follow-up in the external cohort is 29.2 months, and by 60 months only 68 of 664 patients remain at risk; the 60-month calibration and restricted mean survival time estimates in that cohort are consequently based on a small and selected remainder of the sample and are reported for completeness rather than as evidence about five-year performance (Section 3.8). Fifth, the literature survey operates at abstract level and the proportions found are lower bounds. Sixth, the proportional hazards assumption is violated in the internal validation cohort; the concordance index is relatively robust to this, but long-horizon calibration estimates should be interpreted with caution. Seventh, a limitation concerns fairness. Race, ethnicity, socioeconomic status and comparable sociodemographic variables are not recorded in any of the three cohorts, so no formal fairness assessment was possible and the TRIPOD + AI item on known health inequalities is reported as not addressed (Supplementary TRIPOD + AI map). The consequence must be stated plainly rather than left implicit. The development and internal validation cohorts are Chinese, and the external cohort is predominantly North American; the model’s behaviour across sociodemographic groups within either population is entirely unexamined, and nothing in the present results licenses an assumption that discrimination or calibration is equitable across such groups. Subgroup performance is reported by IDH status, 1p/19q status, grade, age band and sex, and it is already unstable across the molecular strata that we can examine, which is a reason to expect rather than discount heterogeneity along axes we cannot examine. A fairness evaluation in cohorts carrying the relevant variables is therefore a prerequisite for any clinical use of this or any comparable signature, and this requirement applies independently of the added-value question that is the subject of this paper. Finally, harmonisation used within-cohort z-scoring, which does not permit a single patient sample to be evaluated in isolation; prospective use would require a fixed reference distribution.

## 5. Conclusions

A leakage-controlled transcriptomic signature showed preserved discrimination across two independent cohorts, with absolute risk estimates that showed no consistent improvement after recalibration. It nevertheless makes no measurable contribution beyond the available clinical reference model containing grade and molecular markers, and it cannot be distinguished in the external cohort from random gene sets drawn from the same candidate pool. These two observations are informative less about the signature itself than about what a concordance index in this range conveys. Most of its discrimination arises from the IDH and grade axis. A survey of 515 records indicates that the field largely lacks the reporting practice required to make these assessments. A concordance index cannot be interpreted without a clinical reference model and a random-signature null distribution, and we propose that both be adopted as a minimum standard in glioma signature studies. For the second of these, one specification is essential if the test is to mean anything: the random sets must be drawn from the same candidate pool that was available to the selection procedure and matched to the size of the signature being tested. A null constructed from the full transcriptome instead of the screened pool consists largely of sets with little prognostic content and will declare almost any screened signature significant; as we show in Section 3.9, the *p*-value in our own external cohort moves from 0.473 to 0.129 as the sampling frame is widened from 100 to 500 genes, with the signature and the test otherwise unchanged. Reporting the frame is therefore as necessary as reporting the *p*-value.

## Figures and Tables

**Table 1:** Demographic, clinical, and molecular characteristics of the development and validation cohorts.

Characteristic	CGGA-693	CGGA-325	TCGA
*n*	404	222	664
Events, *n* (%)	209 (51.7)	138 (62.2)	247 (37.2)
Age, median (IQR)	43 (35–53)	43 (36–53)	44 (34–58)
Male, *n* (%)	231 (57.2)	138 (62.2)	346 (52.1)
WHO II/III/IV	130/141/133	90/47/85	n.a.
IDH-mutant, *n* (%)	197 (53.7)	112 (50.7)	418 (63.0)
1p/19q codeleted, *n* (%)	85 (24.5)	50 (22.8)	n.a.
MGMT methylated, *n* (%)	190 (57.1)	96 (46.2)	n.a.
Median survival, months	63.5	44.7	50.5
Median follow-up, months	75.6	130.2	29.2

n.a.: not available.

**Table 2:** Abstract-level reporting content of 515 glioma signature records.

Criterion	*n*	%
LASSO/penalised selection	400	77.7
Risk score	347	67.4
Nomogram	264	51.3
CGGA validation	242	47.0
Claim of ‘independent prognostic factor’	146	28.3
Calibration	45	8.7
Decision curve/net benefit	23	4.5
Comparison with clinical reference	5	1.0
Brier score	0	0.0
All three	0	0.0

**Table 3:** Calibration metrics. The last column gives the stratum-based approximate measure for comparison.

Cohort	Month	ICI	E50	E90	Slope	O/E	Stratum-Based
CGGA-693	12	0.018	0.021	0.023	1.27	0.99	0.027
CGGA-693	36	0.053	0.059	0.076	1.27	0.95	0.079
CGGA-325	12	0.032	0.023	0.068	1.08	0.90	0.028
CGGA-325	36	0.067	0.079	0.099	1.08	0.84	0.079
TCGA	12	0.020	0.022	0.026	1.18	0.89	0.033
TCGA	36	0.045	0.047	0.080	1.18	0.92	0.064
TCGA	60	0.057	0.058	0.095	1.18	1.02	0.037

## Data Availability

CGGA data can be obtained from cgga.org.cn (accessed on 13 July 2026) and TCGA data through cbioportal.org (accessed on 13 July 2026). The analysis code, the locked model coefficients, the literature survey script, and a verification script confirming that the results are unchanged will be made public upon publication.

## Supplementary Materials

The following supporting information can be downloaded at: https://www.mdpi.com/article/10.3390/diagnostics16172803/s1, Supplementary Methods S1, literature survey protocol; Table S1, locked coefficients of the twenty-gene signature; Table S2, robustness and sensitivity analyses; Table S3, random-signature null distribution; Table S4, sensitivity and mechanism analyses; Table S5, dependence of the null test on the candidate pool; Table S6, abstract-level reporting content of 515 records; TRIPOD+AI reporting map.
