# Supplementary material for: Evaluating a Glioma Transcriptomic Signature Against a Clinical Reference Model and a Random-Signature Null Distribution: A Leakage-Controlled Internal Audit and a Survey of the Field
Source: Diagnostics (Basel). 2026 Aug 31;16(17):2803. doi: 10.3390/diagnostics16172803 (PMC13565182; doi:10.3390/diagnostics16172803)
Supplement: Supplementary file 1 [file diagnostics-16-02803-s001.zip › diagnostics-4530956-supplementary.pdf]

Supplementary Materials

Manuscript: Evaluating a glioma transcriptomic signature against a clinical reference model and a random-signature null distribution: a leakage-controlled internal audit and a survey of the field.

Contents: Supplementary Methods S1 (literature survey protocol); Table S1 (locked signature coefficients); Table S2 (robustness and sensitivity analyses); Table S3 (random-signature null distribution); Table S4 (sensitivity and mechanism analyses); Table S5 (dependence of the null test on the candidate pool); Table S6 (abstract-level reporting content of 515 records); TRIPOD+AI reporting map.

Supplementary Methods S1. Literature survey protocol

Database and date. The Web of Science Core Collection was searched on 9 August 2026 using all editions available through the institutional subscription (Editions: All). No restriction was placed on publication year, language or document type.

Search query. TS=(glioma OR glioblastoma) AND TS=("gene signature" OR "prognostic signature" OR "risk score" OR "gene expression signature") AND TS=(LASSO OR "elastic net" OR nomogram), where TS denotes the Topic field (title, abstract, author keywords and Keywords Plus).

Record selection. The search returned 534 records with no title-level duplicates. Nineteen were excluded because neither glioma nor glioblastoma appeared in the title, abstract or keywords, leaving 515 eligible records published between 2014 and 2026. One record lacked an abstract and was screened on title and keywords alone. Because the document-type field was not included in the exported dataset, the distribution of publication types could not be independently verified; the term record rather than article is therefore used throughout.

Screening procedure. For each record the title, abstract and keywords were concatenated, lowercased and screened with the prespecified regular expressions listed in Table S6. The pattern for comparison with a clinical reference model was deliberately broad and matched any of the following: clinical model, variable, factor, parameter or feature described as alone or only; comparison with clinical, clinicopathological, traditional or conventional information; clinical-only or clinic-only phrasing; added value, incremental value, or information beyond clinical variables; and superiority over or outperformance of a clinical model. No requirement was imposed that the comparison be quantitative, paired, or fitted in the authors' own data.

Interpretation. The survey operates at abstract level. Analyses performed and reported only in the full text were not counted, so all proportions are conservative lower-bound estimates of how often these practices are explicitly reported.

Supplementary Table S1. Locked coefficients of the twenty-gene signature. The model is an elastic-net penalised Cox model with mixing parameter 0.9 and penalty 0.0731, selected by five-fold cross-validation within the development cohort (cross-validated concordance 0.797). Coefficients apply to expression values standardised with development-cohort means and standard deviations. Apparent concordance in the development cohort was 0.819 and 0.801 in the independent internal validation cohort.

Gene	Coefficient	Direction	Description
RGS16	+0.15987	Risk	Regulator of G protein signalling 16
GRB14	+0.19314	Risk	Growth factor receptor bound protein 14
FAM64A (PIMREG)	+0.09105	Risk	Regulator of chromosome condensation
H19	+0.07994	Risk	Imprinted long non-coding RNA
HIST1H2BK	+0.07984	Risk	Histone cluster 1 H2B family member K
IGFBP5	+0.06510	Risk	Insulin-like growth factor binding protein 5
SLC47A2	+0.05849	Risk	Solute carrier family 47 member 2
DRAXIN	+0.04866	Risk	Dorsal inhibitory axon guidance protein
FLNC	+0.04337	Risk	Filamin C
IL13RA2	+0.03661	Risk	Interleukin 13 receptor subunit alpha 2
TNC	+0.03567	Risk	Tenascin C
IGF2BP3	+0.02962	Risk	Insulin-like growth factor 2 mRNA binding protein 3
AC062021.1	−0.02501	Protective	Uncharacterised transcript (clone identifier)
CSDC2	−0.04218	Protective	Cold shock domain containing C2
FAM155A	−0.07133	Protective	Family with sequence similarity 155 member A
WDR86	−0.07155	Protective	WD repeat domain 86
RP11-18I14.10	−0.08897	Protective	Uncharacterised transcript (clone identifier)
KCNJ11	−0.10456	Protective	Potassium inwardly rectifying channel subfamily J member 11
KCNK3	−0.12124	Protective	Potassium two pore domain channel subfamily K member 3
SPOCK2	−0.14253	Protective	SPARC/osteonectin, cwcv and kazal-like domains proteoglycan 2

Supplementary Table S2. Robustness and sensitivity analyses. Unless stated otherwise the quantity reported is Harrell's concordance index. Candidate pool and mixing parameter analyses rebuild the signature under the primary protocol, including the 50-point regularisation path; the number of genes retained for each setting is given in parentheses.

Analysis	Cohort	Value
Proportional hazards (Schoenfeld, rank-weighted)	CGGA-693	p = 0.859
	CGGA-325	p = 0.027
	TCGA	p = 0.066
IPCW concordance (Uno), τ = 36 months	CGGA-325	0.814
IPCW concordance (Uno), τ = 60 months	CGGA-325	0.812
IPCW concordance (Uno), τ = 36 months	TCGA	0.832
IPCW concordance (Uno), τ = 60 months	TCGA	0.826
Harmonisation: within-cohort z-scoring (primary)	TCGA	0.824
Harmonisation: development-cohort scaler	TCGA	0.822
Harmonisation: rank transformation	TCGA	0.821
Candidate pool 100 genes (48 selected)	CGGA-325	0.775
Candidate pool 200 genes (20 selected, primary)	CGGA-325	0.801
Candidate pool 300 genes (39 selected)	CGGA-325	0.796
Candidate pool 500 genes (58 selected)	CGGA-325	0.791
Mixing parameter 0.5 (77 selected)	CGGA-325	0.764
Mixing parameter 0.7 (23 selected)	CGGA-325	0.801
Mixing parameter 0.9 (20 selected, primary)	CGGA-325	0.801
Mixing parameter 1.0 (20 selected)	CGGA-325	0.801
Complete-case analysis (194 patients)	CGGA-325	0.799
Subgroup: IDH-mutant	CGGA-325	0.794
Subgroup: IDH-wildtype	CGGA-325	0.629
Subgroup: 1p/19q non-codeleted	CGGA-325	0.735
Subgroup: WHO grade II	CGGA-325	0.707
Subgroup: WHO grade III	CGGA-325	0.759
Subgroup: WHO grade IV	CGGA-325	0.606
Subgroup: age < 45	CGGA-325	0.846
Subgroup: age ≥ 45	CGGA-325	0.702
Subgroup: male	CGGA-325	0.814
Subgroup: female	CGGA-325	0.794
Subgroup: IDH-mutant	TCGA	0.683
Subgroup: IDH-wildtype	TCGA	0.563
Optimism correction: apparent	CGGA-693	0.819
Optimism correction: optimism	CGGA-693	0.019
Optimism correction: corrected	CGGA-693	0.800

Supplementary Table S3. Random-signature null distribution. Each null comprises 1,000 random twenty-gene sets drawn from the stated frame, excluding the twenty genes of the observed signature. Every set is subjected to the same procedure as the signature: scaled with development-cohort statistics, fitted by an L2-penalised Cox model in the development cohort, and transferred unchanged. p is the proportion of random sets attaining a concordance index equal to or greater than the observed one.

Sampling frame	Cohort	Observed	Null median	Null 95th pct	p
Top 200 candidates (primary)	CGGA-325	0.801	0.777	0.795	0.015
Top 200 candidates (primary)	TCGA	0.821	0.811	0.826	0.154
Top 500 screened genes	CGGA-325	0.801	0.780	0.798	0.027
Top 500 screened genes	TCGA	0.821	0.808	0.825	0.129

Supplementary Table S4. Sensitivity and mechanism analyses. Differences between the signature and the clinical reference model were computed within the same 2,000 bootstrap resamples. Restricted mean survival time (RMST) differences are between risk groups defined by the median of the development cohort, with 1,000-resample bootstrap intervals. The proliferation metagene is built from sixteen canonical proliferation genes, none of which appears in the signature.

Analysis	Quantity	Value
Clinical model including treatment variables	C-index (CGGA-325)	0.799
Difference, signature minus clinical	Δ (95% CI)	+0.001 (−0.032 to +0.036)
Clinical model, diagnosis-time variables only	C-index (CGGA-325)	0.792
Difference, signature minus clinical	Δ (95% CI)	+0.008 (−0.024 to +0.043)
RMST difference at 36 months	CGGA-325	14.2 months (12.0 to 16.3)
RMST difference at 60 months	CGGA-325	31.3 months (27.4 to 34.9)
RMST difference at 36 months	TCGA	14.2 months (12.6 to 15.7)
RMST difference at 60 months	TCGA	28.8 months (25.6 to 31.6)
Signature score versus proliferation metagene	Spearman ρ	0.722
Proliferation metagene alone	C-index (CGGA-325)	0.723
Signature added to metagene	Likelihood ratio χ^2	67.3
Genes of top 500 absent from TCGA	n	51

Supplementary Table S5. Dependence of the random-signature null test on the candidate pool. **Panel A**: the signature is rebuilt from scratch within each pool under the identical protocol and tested against a null drawn from that same pool, with random sets matched to the size of the resulting signature. **Panel B**: the primary 20-gene signature and the random set size are held fixed and only the frame from which random sets are drawn is varied. All null distributions comprise 1,000 random sets; p is the proportion attaining a concordance index equal to or greater than the observed value.

Panel A. Matched pool

Pool	Genes selected	C (CGGA-325)	Null median (325)	p (325)	C (TCGA)	Null median (TCGA)	p (TCGA)
Top 100	48	0.750	0.758	1.000	0.812	0.818	0.983
**Top 200 (primary)**	**20**	**0.801**	**0.777**	**0.015**	**0.821**	**0.811**	**0.154**
Top 500	58	0.774	0.763	0.185	0.785	0.798	0.748

Panel B. Frame effect, signature and set size fixed at 20 genes

Sampling frame	Null median (325)	p (325)	Null median (TCGA)	p (TCGA)
Top 100	0.775	0.005	0.820	0.473
**Top 200 (matched to selection pool)**	**0.777**	**0.015**	**0.811**	**0.154**
Top 500	0.780	0.027	0.808	0.129

Observed concordance indices in this table were computed under the null protocol—the gene set is refitted by L2-penalised Cox regression in the development cohort and transferred unchanged—so that the observed value and the null distribution are produced by an identical procedure. They therefore differ slightly from the values in Table S2, which were computed under the primary locked elastic-net protocol.

Supplementary Table S6. Abstract-level reporting content of 515 glioma signature records indexed in the Web of Science Core Collection. Patterns were prespecified and applied to the concatenated title, abstract and keyword text; the full pattern for clinical reference comparison is given in Methods S1. Derived rows were computed by combining the criteria above them.

Criterion	n	%	Match terms
LASSO or equivalent penalised selection	400	77.7	lasso, least absolute shrinkage, elastic net
Risk score	347	67.4	risk score
Nomogram	264	51.3	nomogram
CGGA validation	242	47.0	cgga
Claim of 'independent prognostic factor'	146	28.3	independent prognostic
Calibration	45	8.7	calibration
Concordance index	33	6.4	c-index, concordance index
Decision curve or net benefit	23	4.5	decision curve, net benefit, clinical utility
Comparison with clinical reference	5	1.0	see Methods S1
Brier score	0	0.0	brier
Discrimination reported, calibration absent	17	3.3	derived
Discrimination reported, clinical comparison absent	33	6.4	derived
All three reported together	0	0.0	derived

Supplementary File: TRIPOD+AI reporting map

Manuscript: Evaluating a glioma transcriptomic signature against a clinical reference model and a random-signature null distribution: a leakage-controlled internal audit and a survey of the field.

Purpose and scope of this document

TRIPOD+AI (Collins GS, Moons KGM, Dhiman P, et al. BMJ 2024;385:e078378) is the current reporting guideline for clinical prediction model studies that use regression or machine learning methods and supersedes TRIPOD 2015. It comprises 27 main items with 10 subitems, organised into title and abstract, introduction, methods, results, discussion, and other information.

The table below maps the reporting requirements of TRIPOD+AI to the locations in the present manuscript where each is addressed. The reporting elements are stated in summary form rather than reproduced from the checklist; the official numbered checklist form should be obtained from www.tripod-statement.org and the locations transferred onto it at submission. The map is intended as a transparent reporting aid, not as a claim that every requirement could be satisfied from retrospective, publicly available data. Requirements that were not met, not applicable, or not possible with the available data are stated explicitly rather than omitted.

Reporting map

Reporting element (summarised)	Where addressed	Status and comments
TITLE AND ABSTRACT		
Study type, target population, predicted outcome identified in title	Title page	Reported. The title states that a glioma transcriptomic signature is evaluated against a clinical reference model and a random-signature null distribution.
Structured abstract covering objectives, data, methods, performance, conclusions	Abstract	Reported. Objectives, three cohorts, sample sizes and events, model class, discrimination with confidence intervals, calibration index and conclusions are given.
INTRODUCTION		
Healthcare context and rationale, with reference to existing models	Section 1, paragraphs 1–4	Reported. Prognostic setting stated; thirteen existing glioma signatures cited; the random-signature literature is summarised.
Target population and intended use of the model in the care pathway	Section 1 paragraph 7; Section 4.2	Partially reported. Intended use is framed as a prognostic substitute where clinical and molecular annotation is unavailable; the manuscript states that prospective testing would be required before any care-pathway use.

Known health inequalities between sociodemographic groups	Section 4.7	Not reported, with consequences stated. Race, ethnicity and socioeconomic variables are unavailable in all three cohorts. Development and internal validation are Chinese and external validation is predominantly North American. Section 4.7 states the direct consequence: fairness and generalisability across sociodemographic groups remain unexamined, and evaluation in cohorts carrying these variables is a prerequisite for any clinical use.
Study objectives, and whether development, evaluation or both	Abstract; Section 1 paragraph 7	Reported. Three objectives stated; the study covers development and both internal and external evaluation.
METHODS—DATA AND PARTICIPANTS		
Sources of data, separately for development and evaluation	Section 2.1	Reported. CGGA mRNAseq_693 (development), CGGA mRNAseq_325 (internal validation), TCGA LGG+GBM (external validation), with source citations.
Eligibility criteria and setting	Section 2.1	Reported. Primary tumours only; recurrent and secondary cases excluded; patients without survival time or event status excluded.
Study dates and follow-up	Table 1	Reported. Median follow-up given for each cohort (75.6, 130.2 and 29.2 months). Accrual dates are governed by the source repositories and are not set by the present study.
Data preparation, including transformation and harmonisation	Section 2.2	Reported. log2(x+1) transformation, expression filtering, within-cohort z-scoring for the external cohort, and the prespecified rule for signature genes unavailable in a target cohort.
METHODS—OUTCOME, PREDICTORS, SAMPLE SIZE		

Outcome definition and time horizon	Section 2.1; Section 2.5	Reported. Overall survival; calibration and utility assessed at 12, 24, 36 and 60 months.
Predictors: definition, measurement, and blinding to outcome	Sections 2.2–2.4	Reported. Predictors are RNA-sequencing expression values and routinely recorded clinical variables. Screening and selection were performed in the development cohort only; validation cohorts were not examined during model building.
Sample size justification	Section 4.7	Not prespecified. The manuscript states that the number of events in the development cohort falls below recommended minimum sample size criteria for the number of candidate variables, and that penalised regression limits but does not eliminate this risk.
Missing data handling	Section 2.4; Section 3.8	Reported. Median imputation with missingness indicators, with medians computed in the development cohort only. A complete-case sensitivity analysis is reported (0.799 versus 0.801).
METHODS—ANALYSIS		
Model type and model-building steps	Sections 2.3–2.4	Reported. Univariate Cox score-test screening with Benjamini–Hochberg control, top 200 candidates, elastic-net penalised Cox with mixing parameter 0.9 and cross-validated penalty.
Approach to hyperparameter tuning and internal validation	Section 2.4; Section 3.8	Reported. Five-fold cross-validation within the development cohort; bootstrap optimism correction reported (apparent 0.819, optimism 0.019, corrected 0.800).
Measures taken against information leakage	Section 2.4	Reported. Imputation medians, scaling statistics, candidate pool and penalty parameter derived from the development cohort only; final model locked to file before validation.

Class imbalance handling	—	Reported. The outcome is time-to-event, so the item does not apply in the form defined for classification tasks; event fractions across cohorts are 37-62%. Incidence at a fixed horizon can nonetheless be imbalanced—in the external cohort 84 of 664 patients had experienced the event by 12 months and 190 by 36 months—and the item is therefore addressed rather than dismissed. No resampling, class weighting or synthetic oversampling was applied, which is the appropriate choice for a survival model: resampling distorts the risk-set structure on which partial likelihood estimation depends and would invalidate the absolute risk estimates that the calibration analysis assesses. Overfitting arising from the high-dimensional setting was addressed instead by elastic-net penalised Cox regression with the penalty selected by cross-validation within the development cohort.
Model output and how risk is presented	Section 2.4; Supplementary Table S1	Reported. A linear predictor from locked coefficients, converted to absolute survival probability through a Breslow baseline hazard estimated in the development cohort.
Performance measures: discrimination, calibration, clinical utility	Section 2.5	Reported. Harrell and inverse-probability-of-censoring-weighted concordance, landmark AUC, integrated calibration index with E50, E90, calibration slope and observed-to-expected ratio, and decision curve analysis.
Model updating or recalibration	Section 2.5; Section 3.6	Reported. Slope and baseline hazard recalibration was attempted in the

		external cohort with coefficients held fixed; results reported for 12 to 60 months.
Fairness and subgroup performance	Section 3.8	Partially reported. Performance is reported by IDH status, 1p/19q status, WHO grade, age band and sex. Race, ethnicity and socioeconomic variables are unavailable, so a formal fairness assessment was not possible.
RESULTS		
Participant flow and characteristics	Section 3.1; Table 1	Reported. Cohort sizes, events, age, sex, grade, molecular markers, treatment and follow-up for all three cohorts.
Model specification: predictors and coefficients	Supplementary Table S1	Reported. All twenty genes with coefficients, direction, mixing parameter, penalty value and cross-validated concordance.
Performance with uncertainty	Sections 3.4–3.7; Tables 2–3; Figures 2–6	Reported. Concordance with 2000-resample bootstrap intervals, paired between-arm differences, calibration metrics at four horizons, decision curves and the random-signature null distribution.
Results of model updating	Section 3.6	Reported. Integrated calibration index before and after recalibration at each horizon; no consistent improvement observed.
DISCUSSION		
Interpretation in the context of objectives and existing evidence	Sections 4.1–4.5	Reported. Dissociation between independence and added value; comparison with a contemporaneous study using the same cohorts; interpretation against the random-signature literature.
Limitations	Section 4.7	Reported. Six limitations, including unequal clinical annotation across cohorts, absence of performance status and extent of resection, sample size,

		short external follow-up, abstract-level literature survey and proportional hazards violation.
Usability and implications for practice or research	Sections 4.2, 4.5; Section 5	Reported. The manuscript states that the signature is not proposed for clinical deployment and proposes three evaluation criteria as a minimum standard for the field.
OTHER INFORMATION		
Study registration and protocol availability	—	Not registered. This is a retrospective secondary analysis of public data and was not prospectively registered; no protocol was deposited.
Funding	Declarations	To be completed by the authors at submission.
Competing interests	Declarations	Reported. The authors declare no competing interests.
Data availability	Data and code availability	Reported. CGGA and TCGA are publicly available with sources named.
Code availability	Data and code availability	Reported. Analysis code, locked model coefficients, literature survey script and the reproducibility verification script will be made public upon publication.
Patient and public involvement	—	None. No patients or members of the public were involved in the design, conduct or reporting of this study, which used only de-identified public data.
Ethical approval	Cover letter; Declarations	Reported. Institutional review board approval was not required for secondary analysis of publicly available de-identified data.

Summary of requirements not met

Five requirements are not met and are declared as such: known health inequalities between sociodemographic groups could not be described because the relevant variables are unavailable in all cohorts; a formal fairness assessment was not possible for the same reason, although subgroup performance is reported by IDH status, 1p/19q status, grade, age and sex—the manuscript states in Section 4.7 that this leaves fairness and generalisability across diverse populations unexamined and that addressing it is a prerequisite for clinical use; the sample size was

not prespecified and falls below recommended criteria, as stated in the limitations; the study was not prospectively registered and no protocol was deposited; and there was no patient or public involvement, the study having used only de-identified public data.
